# Finite Element Analysis of Soft-Pad Moldless Stamping of Bistable Circular Micro Shells

**DOI:** 10.3390/mi16030294

**Published:** 2025-02-28

**Authors:** Mark M. Kantor, Asaf Asher, Rivka Gilat, Skava Krylov

**Affiliations:** 1 Department of Civil Engineering, Faculty of Engineering, Ariel University, Ariel 40700, Israel; mark.m.kantor@gmail.com; 2 Faculty of Engineering, School of Mechanical Engineering, Tel-Aviv University, Tel Aviv 69978, Israel; asafasher83@gmail.com (A.A.); krylov@tauex.tau.ac.il (S.K.)

**Keywords:** MEMS/NEMS, micro cold forming, thin films, bistable curved plates, inelastic deformation, nonlinear foam

## Abstract

Bistable microstructures are promising for implementation in many mictroelectromechanical system (MEMS)-based applications due to their ability to stay in several equilibrium states, high tunability and unprecedented sensitivity to external stimuli. As opposed to the extensively investigated one-dimensional curved beam-type devices of this kind, microfabrication of non-planar two-dimensional bistable structures, such as plates or shells, represents a remarkable challenge. Recently reported by us, a new moldless stamping procedure, based on pressing a soft stamp over a thin suspended metallic film, was demonstrated to be a feasible direction for the fabrication of initially curved micro plates. However, reliable implementation of this fabrication paradigm and its further development requires better understanding of the role of the process parameters, and of the effect of both the plate and the stamp material properties on the shape of the formed shell and on the postfabrication residual stresses, and therefore on the shell behavior. The need for an appropriate choice of these parameters requires the development of a systematic modeling approach to the stamping process. Here, we report on a finite element (FE)-based methodology for modeling the processing sequences of a successfully fabricated aluminum (Al) micro shell of realistic geometry. The model accounts for the elasto-plastic behavior of the plate material, the nonlinear material behavior of the foam and the contact between them. It was found that the stamping pressure and the plate material parameters are the key parameters affecting the residual shell curvature as well as its shape. Consistently with previously presented experimental results, we show that the fabrication procedure partially relieves the prestresses emerging during preceding fabrication steps, leaving a nontrivial distribution of residual stresses in the formed shell. The presented analysis approach and results provide tools for designers and manufacturers of systems including micro structural elements of shell type.

## 1. Introduction

The usage of initially curved thin-walled 2D/3D micro and nano structural elements, for various applications such as sensing, actuating and filtering in a variety of fields including electronics, fluidics, mechanics, acoustics and optics, attracts continuous interest [1,2,3]. An especially appealing feature of such elements is bistability, namely the existence of two different equilibrium states, characterized by distinct structural configurations, corresponding to the same loading. The utilization of bistabilty in 1D curved beams has been extensively investigated [4,5,6,7], and its possible emergence in micro shells has been theoretically and experimentally demonstrated [8,9,10]. However, to facilitate the widespread use of 3D thin micro shells, efficient technologies for their microfabrication should be developed.

While traditional lithography-based processes, originally developed for planar configurations, are suitable for the fabrication of flat and curved 1D beams and 2D flat micro plates, their implementation for the fabrication of curved plates and shells is challenging. Hence, other directions that might be more suitable for shaping 3D shell structures have been considered, including high-temperature glass blowing [11] and additive manufacturing techniques [12]. A possible adaption of the cold sheet metal forming approach, widely used at the macro scale, was also examined [13,14]. Recently, a new sheet cold forming procedure, which is potentially suitable for wafer-level fabrication of thin-walled micro shells, has been suggested [9]. It was employed to fabricate aluminum circular bell-shaped micro shells with a thickness of ≈0.4 μm and radii varying between ≈100 μm and ≈700 μm. Bistable behavior and high frequency tunability up to 50% of the resulting structures under mechanical and electrostatic loading were experimentally demonstrated [9,10], indicating that the moldless soft-pad stamping approach can generate curvatures larger than the bistability threshold. However, as reported in [9], the success of the stamping process and its outcome depends on numerous parameters, including the soft stamp and the plate material mechanical parameters, stamping pressure, flat plate characteristics and their right combination.

At the macro scale, numerical analyses of common sheet forming processes have been conducted in order to improve product quality while reducing production time and cost [15,16]. Currently, the FE method forms the main tool for large-scale forming simulations [17]. A few applications of the FEM for the analysis of micro sheet forming can also be found [18,19].

**Figure 1:**
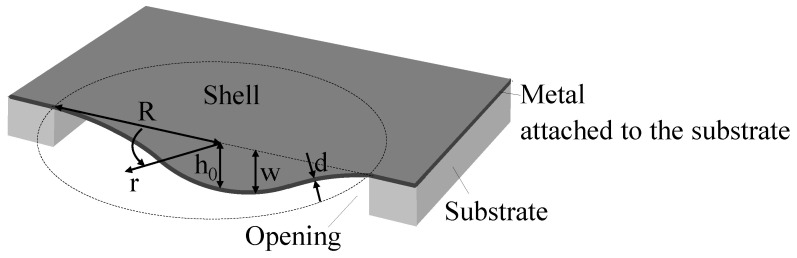
Schematic geometry of the micro shell.

In the present work, an FE-based model of the recently suggested modified cold forming process is established. The model is specifically adapted to enable the analysis of the new moldless stamping procedure, which differs from common cold forming approaches widely implemented at the macro scale. First, it is based on a self-spreading soft punch in the absence of a rigid mold, yielding a final shape that is not dictated by a mold but rather depends on the various process parameters. Consequently, the modeling should account for factors such as foam–plate interaction, foam compressibility and nonlinearity. Second, at the micro scale, residual stresses that emerge during the fabrication process may significantly affect the subsequent operation of the formed element [5,10,20]. Specifically, information about the residual stresses in the post-forming state is important when considering the potential use of the presently discussed bistable circular shells as (resonant) sensors or switches. Note that the fabrication-related residual stresses in the flat plates, which are the starting point of the forming, may have an influence on the stamping itself. Third, while in the macro forming scenario the stamped sheet can slide inside the mold, in the case of microfabrication the flat plate boundaries are fixed (since the non-suspended part of the film, within the area outside the opening in the substrate, is attached to the substrate, Figure 1). Moreover, the thickness-to-radius ratio of the stamped micro plate is smaller than that at the macro scale. As a result, while the macro stamping is essentially bending-dominated, the micro stamping is stretching-dominated, and is therefore accompanied by a higher level of nonlinearity. These special aspects are handled by the FE analysis of the stamping procedure presented in this work, with the goal of numerically investigating the effect of various parameters and their combinations on the forming process and on the resulting shell shape and state of residual stresses. To relate the real process to its simulation, the numerically predicted profiles were compared to the profile of an actually stamped micro shell. The results of the present study can be employed to improve the design of the stamping procedure, aimed at providing a desired shell geometry.

## 2. The Fabrication Process

**Figure 2:**
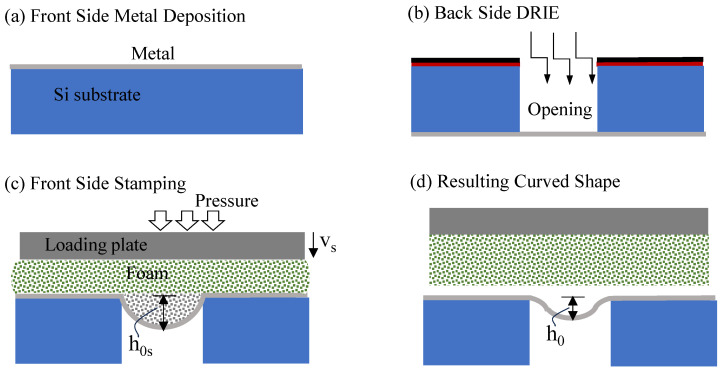
Fabrication process flow [9]. (**a**) Structural metal layer deposition on the front side of the Si wafer. (**b**) Opening of a cavity in the back side of the substrate by deep reactive ion etch with the front-side metal layer serving as the etch stop. (**c**) FS soft-foam self-molding stamping. (**d**) Residual curved shape.

Before presenting the numerical model developed in this work, it is instructive to briefly review the approach used in [9] to fabricate axisymmetric bistable micro shells with a circular horizontal projection of radius *R*, central height $h_{0}$ and thickness *d*, as shown in Figure 1. First, free-hanging circular, flat, thin Al sheets are prepared by the following process. The Al metal layer is deposited (sputtered) on a silicon wafer, Figure 2a. A circular opening is deep reactive ion-etched (DRIE) in the substrate, with the metal film serving as the etch stop, Figure 2b. This results in a suspended circular metal flat plate clamped along its perimeter. The final shape of the shell is achieved by pressing a soft punch, consisting of a foam layer, over the entire area of the Si die, including the thin suspended metal sheet, Figure 2c. Since the foam layer is not patterned and covers the entire area of the substrate, including the cavity, there is no need for a preshaped three-dimensional punch or mold, the need for alignment with the openings in the substrate is eliminated and multiple devices can be processed simultaneously.

Pressure is applied through the vertical displacement of a stiff loading plate. The compressed foam transfers stresses to the metal layer surface through the natural contact between them. This pushes the suspended circular plate into the cavity while inducing elastic and plastic deformation in the metal sheet, giving it an instantaneous shape similar to a spherical cap with a central height $h_{0s}$. Upon the release of the stamp, the reversible elastic part of the plate deformation is released, and a residual bell shape with a central height $h_{0}$ is obtained, Figure 2d.

Our fabrication experiences indicate that to achieve the desired structural configuration while avoiding film rupture, the process parameters should be appropriately chosen. The extent of $h_{0s}$ depends on the stamping pressure, dominated by the interplay between the peak stamping downward displacement of the loading plate, $v_{s}$, and the stamp stiffness, as well as on the stamped plate parameters. On one hand, for a nonzero $h_{0}$ to emerge, the peak stamping height $h_{0s}$ should be above a certain threshold value. On the other hand, if $h_{0s}$ is too high, the stamping may result in a failure of the plate material and rupture. The trade-off between the minimal and the maximal values of $h_{0s}$ is one of the key difficulties of the process: the appropriate choice of this range assures feasible fabrication and is one of the goals of the here-presented FE modeling, which provides insight into the various parameters’ effect on the resulting shell shape and its state of residual stresses.

The predicted resulting shell shapes were compared to the measured profile of an actually fabricated micro shell [9]. This specific actual bell-shaped shell with central elevation of $h_{0}$≈ 46 μm had been stamped out of a thin aluminum circular flat plate of nominal thickness d≈ 0.4 μm and nominal radius R≈ 425 μm.

## 3. Material Models

The FE model to be established should reflect the behavior of two deformable bodies, the soft foam punch and the circular clamped metal sheet, and their interaction. Thus, suitable constitutive relations for both the stamp foam and the plate metallic material should be adopted.

### 3.1. Foam Material Model

**Figure 3:**
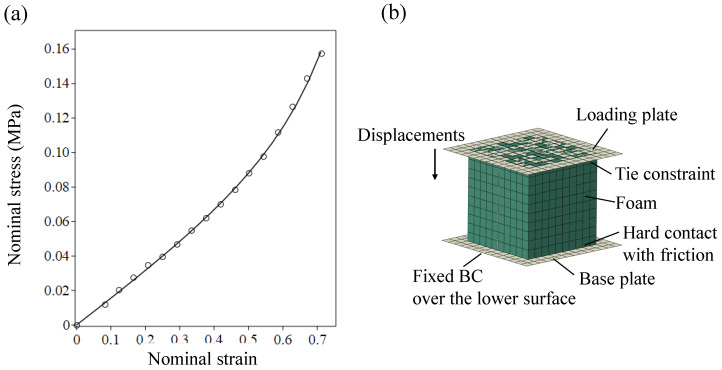
(**a**) Foam behavior as extracted from a compression test (circles) and from its adjusted FE analysis (line). (**b**) FE model of the foam compression test.

In the framework of the development of the fabrication approach reported in [9], various types of soft materials were examined for stamp function. Since the manufacturer data of the material were not available, to characterize the foam that was found the most suitable, a simple unidirectional compression test was conducted on three specimens made of that material, polyethylene. The adjustment of a testing technique that is suitable for a specific foam designated for a specific application is a challenging task [21] and could be the subject of a separate research project. Aiming at just estimating the material behavior, the following simple experimental approach was adopted. A cubic specimen, of 1 cm long edges, with its top surface glued to a stiff plate, was placed on a weighing scale. The top loading plate was pushed down with a micrometer stage, while monitoring its displacements. The measurement of the lateral expansion, with a caliper of 10μm resolution, indicated a close to zero lateral size change under various applied vertical displacements. Nominal stress values were defined through the division of the measured force by the original undeformed cross-section area of the specimen, and nominal strains were obtained by dividing the cube height contraction by its original length. The averaged results of these tests are presented by the solid circles in Figure 3a. Note that the foam samples used in both the material test and the stamping procedure shared similar geometry and loading conditions.

To extract three-dimensional constitutive relations from the results of the uniaxial tests, ref. [22] was used with the material model of [23], which is suitable for compressible foams. In this framework, the strain energy has the following general form:

(1) $$\begin{array}{c} \begin{array}{c} U=\sum_{i=1}^{N}\frac{2\mu_{i}}{\alpha_{i}^{2}}\left[\lambda_{1}^{\alpha_{i}}+\lambda_{2}^{\alpha_{i}}+\lambda_{3}^{\alpha_{i}}-3+\frac{1}{\beta_{i}}\left(J^{-\alpha_{i}\beta_{i}}-1\right)\right], \end{array} \end{array}$$

where $\lambda_{k}$, k=1,2,3 are the principal stretches, $J=\lambda_{1}\lambda_{2}\lambda_{3}$ is the volumetric change and *N* is the number of terms in the series expansion. The material parameters $\mu_{i}$ are related to the shear response, $\beta_{i}$ determine the degree of compressibility (Piosson functions defined with respect to the logarithmic strains [24]) and $\alpha_{i}$ are additional material parameters. The principal (2nd Piola–Kirchoff) stress is given by $S_{k}=\frac{\partial U}{\partial E_{k}}=\frac{\partial U}{\partial \lambda_{k}}$, k=1,2,3, where $E_{k}=\lambda_{k}-1$ is the Lagrangian finite strain. This implies that the initial shear modulus $\mu_{0}$ is given by $\mu_{0}=\sum_{i=1}^{N}\mu_{i}$ and the corresponding bulk modulus is $K_{0}=\sum_{i=1}^{N}2\mu_{i}\left(\frac{1}{3}+\beta_{i}\right)$. Thus, for a stable initial state $\beta_{i}>-\frac{1}{3}$.

The results acquired from the simple compression tests alone are not sufficient for defining the numerous material parameters required for the material model application. Thus, some of them were assumed, while the rest were obtained through a fitting process. Specifically, as the rough measurements of lateral deformations imply a constant vanishing Poison effect, the Poisson ratio was assumed to be ν=0.01. In the absence of bulk test data, and under the experimentally based assumption of a strain-independent Poisson’s effect, the Poisson functions become $\beta_{i}=\beta =\frac{\nu }{1-2\nu }$. The rest of the parameters $\mu_{i}$ and $\alpha_{i}$ with i=1⋯N were determined by means of a material evaluation tool implemented in Abaqus CAE and using least square fit, within trial-and-error loops for various *N* values. Practically, the usage of the Abaqus CAE material evaluation tool requires only the specification of Poisson’s ratio and the uniaxial stress–strain relation. The adopted parameters, which form one optional set of parameters, are listed in Table 1.

To support the validity of the adjusted material model and parameters, an FE model of the compression test was conducted, as shown in Figure 3b. The foam specimen was modeled by 35486 3D solid full-integration eight-node brick elements with three linear interpolation functions (C3D8), which enable easier treatment of contact problems. The element number was chosen through a convergence test. The base plate and the loading plate were modeled by rigid shell elements (R3D4). The normal-to-surface interaction between the foam bottom and its support was modeled by a “hard” contact, which minimizes the penetration of the slave surface into the master surface. The tangential interaction was characterized by a friction coefficient, which equals 0.1, as examination of the effect of this parameter on the analysis results showed small sensitivity with respect to variation between 0 and 1. The interaction between the foam top and the loading plate to which it is glued was modeled using a “tie constraint”, guaranteeing continuity of the normal displacements and vanishing tangential displacements, namely no sliding. The loading simulation was carried out by application of displacements to the loading plate, similar to those measured in the experiment. The predictions of the 3D FE analysis compare well with the test results, as exhibited in Figure 3a.

### 3.2. Metal Material Model

The considered actual micro shell was stamped out of a ≈400 nm thick aluminum layer that was deposited by sputtering [9]. The intensive research dedicated to the mechanical properties of thin metallic films indicates that they can vary significantly, depending on the layer thickness, fabrication methods, fabrication process parameters, annealing history and interaction with other materials ([25,26,27]). As the thin sheet material had not been experimentally characterized, it was assumed to be an isotropic elasto-plastic material represented by the von Misses yield criterion with the associated plastic flow rule. Material parameters within the range reported in the literature were adopted, and their influence on the modeled micro forming process and results was examined. Specifically, a Poisson ratio of 0.3 was adopted, with various values of the Young modulus [25,26,28,29,30,31,32,33]. For simplicity, which can be useful for illuminating the role of the plate material characteristics in the stamping process, isotropic linear hardening was assumed. It was defined by setting two points on the inelastic part of equivalent stress–strain curves, specifically the equivalent yield stress corresponding to zero equivalent plastic strain $\sigma_{e}\left({\bar{\epsilon }}^{pl}=0\right)=\sigma_{Y}=130$ MPa and the equivalent ultimate stress $\sigma_{e}\left({\bar{\epsilon }}^{pl}=0.14\right)=\sigma_{U}=390$ MPa [34,35].

## 4. Finite Element Model

**Figure 4:**
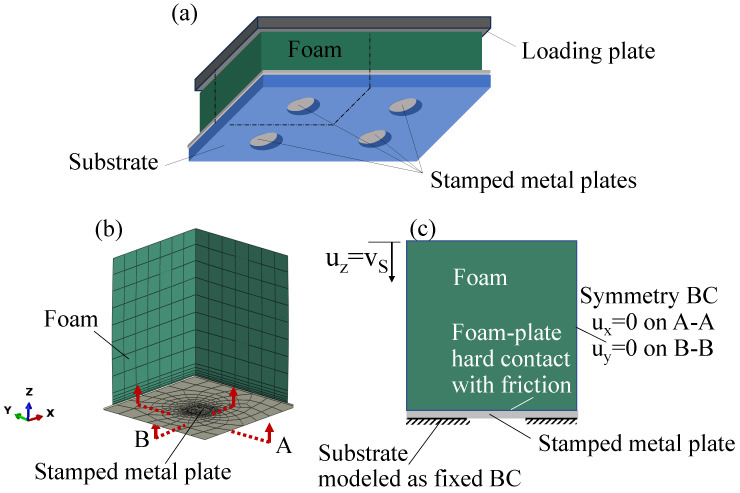
(**a**) Schematic description of the actually stamped device containing four suspended plates (see Figure 5 in [9]). (**b**,**c**) FE model for the bistable micro shell fabrication process analysis. The Si substrate supporting the circular free-hanging part of the metal sheet and the loading plate are not shown in (**b**), and are represented in the cross-section (**c**) by the bottom fixed boundary conditions and the top uniform applied displacement, correspondingly.

To analyze the fabrication process of bistable micro shells, a nonlinear FE model, described in Figure 4b,c, was built. The model consisted of a thin metal plate and a foam stamp, and represented a quarter of the actually stamped die, which contained four suspended circular plates, as schematically shown in Figure 4a (see Figure 5 in [9]).

The stamp with the above-described behavior of highly compressible elastomeric material was modeled by 3D solid full-integration eight-node linear brick elements (C3D8). The 400 nm thick metal sheet, used for stamping the actual micro shell referred to in the present study, was modeled using four-node shell elements (S4) with finite membrane strains and the above-described elasto-plastic constitutive law. This type of element was used due to its suitability for contact problems [22], with 4332 elements of varying size (smaller in the stamping area and larger far from the stamping area) to guarantee convergence. The “hard” contact algorithm was used for describing the normal-to-surface interaction between the mutually deforming stamp and metal sheet. Since the substrate, supporting the thin film over the regions around the cavity, is much stiffer than the other components of the setup, the interaction between the substrate and metal plate was not modeled but was rather represented by fixed boundary conditions.

The analysis of the cold forming procedure consists of two steps, Figure 2c,d. During the first one, displacement constraints are quasistatically imposed on the upper face of the foam stamp. During the second step, foam top displacements are quasistatically decreased until its lower surface is detached from the stamped shell upper surface.

## 5. Results and Discussion

The parametric study was mainly focused on the influence of the stamping pressure, which can be related to the maximum stamping displacement of the foam top, $v_{S}$, and on the stamping foam–plate friction, as well as on the influence of the metal sheet material stiffness *E* and its geometry, represented by its aspect ratio d/R, on the resulting shell. As thin metal film deposition, the fabrication steps described in Figure 2a,b, may result in a nonvanishing state of stresses in the flat, thin, free-hanging metal sheet [36], and as the presence of such stresses in the actually fabricated flat plates was experimentally demonstrated in our recent work [10], the effect of such a prestress, $\sigma_{0}$, was also considered. Specifically, the influence of these various parameters on the obtained residual shell geometry, characterized by its shape w(r) and shallowness, which is represented by $h_{0}$ (Figure 1), and on the post-stamping internal residual stresses, $\sigma_{rr}\left(r\right)$, $\sigma_{\theta \theta }\left(r\right)$, was investigated. Two intermediate process parameters, the foam-to-metal-sheet peak contact pressure distribution $\sigma_{c}\left(r\right)$ and the peak stamping shell height $h_{0_{S}}$ (Figure 2), were also inspected.

**Figure 5:**
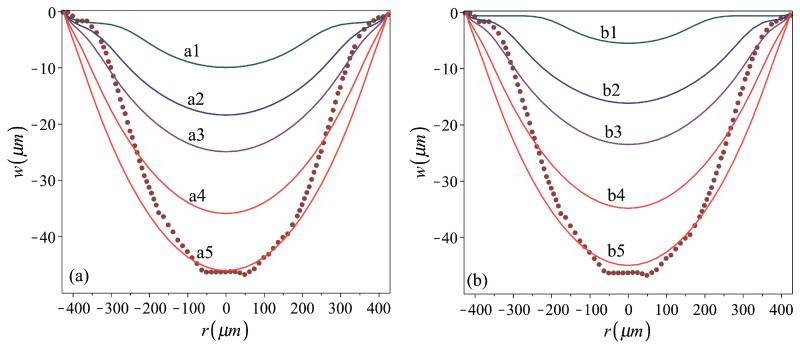
Residual profiles of shells stamped out of (**a**) stress-free plates, cases a1–a5, and (**b**) prestressed to $\sigma_{0}=130$ MPa plates, cases b1–b5. Stamping and material parameters for the analyzed cases are given in Table 2. The dark red dotted line represents the fabricated shell profile [9].

*Role of the stamping pressure*. The effect of the stamping peak pressure on the residual profile and shallowness of shells stamped out of stress-free flat plates with $\sigma_{rr_{0}}=\sigma_{\theta \theta_{0}}=\sigma_{0}=0$ is illustrated in Figure 5a, which is related to the middle column of Table 2. The stamping peak pressure can be reflected by the peak displacement $v_{S}$ or by the peak stamping height $h_{0_{S}}$. The table shows that the relation between these two measures is about the same for all the considered cases, which share a specific combination of foam material and stamped sheet material and geometry. The relation $h_{0}/h_{0_{S}}$ increases with increasing loading pressure, which induces larger inelastic irreversible deformation.

**Figure 6:**
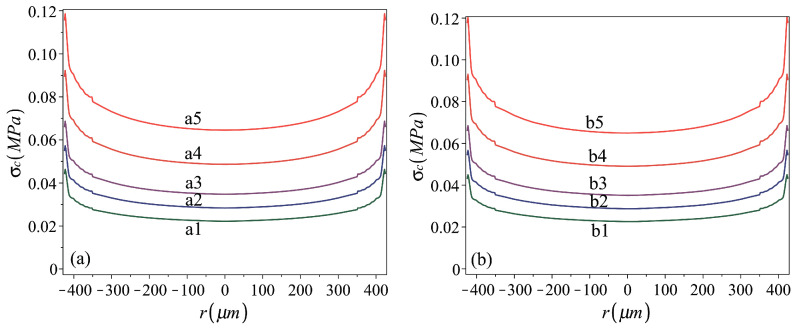
Foam-to-metal-sheet contact peak pressure distribution during stamping of (**a**) stress-free plates, cases a1–a5, and (**b**) prestressed to $\sigma_{0}=130$ MPa plates, cases b1–b5. Stamping and material parameters for analyzed cases are given in Table 2.

The corresponding foam-to-metal-sheet peak contact pressure distributions for a1–a5 cases are shown in Figure 6a, exhibiting a mild variation in stamping pressure over most of the stamped sheets. In all considered cases, under the peak stamping pressure an instantaneous cap shape, with positive curvature almost all over the plate area, is obtained (Figure 2c). This indicates a membrane (stretch- rather than bending-dominated) behavior under the stamping pressure [37,38], implying that rough approximations for $h_{0_{S}}$ can be obtained by membrane theory analysis of uniformly pressed plates [38], while using the elasto-plastic reduced material stiffness.

The curves presented in Figure 5a show that smaller stamping pressures yield shells of a residual bell shape, with negative curvature in a region near the perimeter and positive curvature within the central region (i.e., a3). Under larger stamping stresses, shells of a residual cap shape (i.e., a5) are obtained.

**Figure 7:**
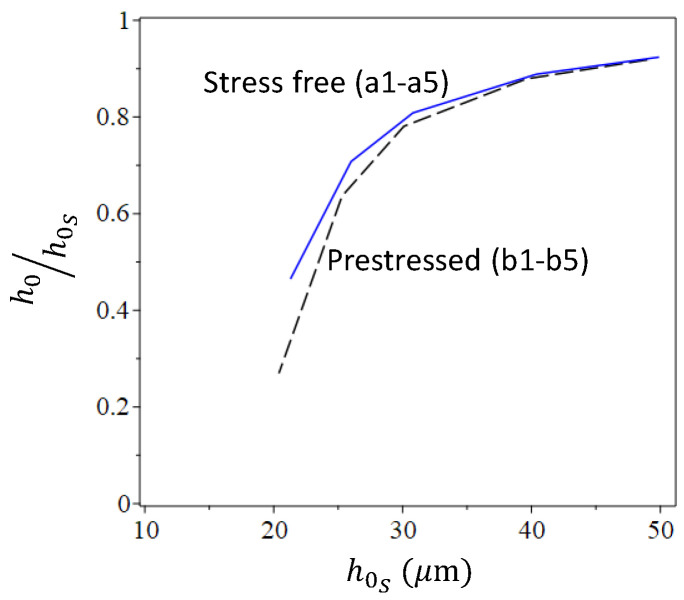
Effect of prestress on the stamping process and results as expressed by the variation in residual-to-peak stamping heights, with the variation in peak stamping height reflecting the stamping pressure. Stamping and material parameters for simulated cases are given in Table 2.

*Role of the membrane prestresses*. Cases corresponding to the above-considered ones, while including uniformly distributed equal radial and circumferential prestresses $\sigma_{rr_{0}}=\sigma_{\theta \theta_{0}}=\sigma_{0}=130$ MPa, are presented in the third column of Table 2 and in Figure 5b and Figure 6b. Curves showing the variation in the residual-to-peak stamping height relations $h_{0}/h_{0_{S}}$ with the increase in the stamping pressure (which can be represented by either $v_{S}$ or $h_{0_{S}}$) for the two groups are presented in Figure 7. The curves indicate that there is a threshold value of $h_{0_{S}}$, guaranteeing nonzero $h_{0}$, that this threshold value is larger in the presence of prestress and that the effect of the prestress is reduced when increasing the stamping pressure, namely when stamping shells of larger residual heights. In such a case, the irreversible deformation induced during stamping first increases the plate size to make it cover the opening while flat and stress-free, while only the development of further deformation enables the nonplanar form. Nevertheless, the difference between the residual deformation of prestressed and initially stress-free cases becomes less significant as the stamping pressure is increased, inducing larger inelastic deformations.

**Figure 8:**
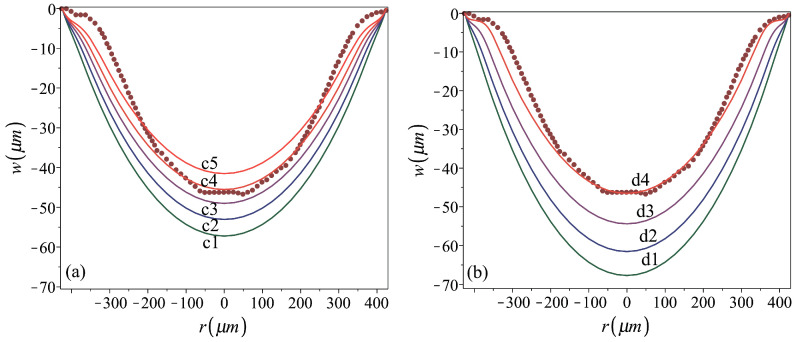
Sensitivity of the residual profile to Young’s modulus of (**a**) stress-free plates, cases c1–c5, and (**b**) prestressed to 130 MPa plates, cases d1–d4. Fabrication and material parameters for the analyzed cases are given in Table 3. The dark red dotted line represents the stamped shell profile [9].

*Role of Young’s modulus*. Aiming at the fabrication of shallow shell elements exhibiting bistable behavior, the prime geometric factor that is of interest is the shell curvature, as expressed in term of its residual center height, $h_{0}$ (with respect to its radius) [39]. Yet, the comparison between the analysis results and the result of the stamping experiment, namely the measured actual profile of the fabricated shell, indicates discrepancy in terms of shapes near to the plate clamping boundaries. While the actual fabricated shell is of a bell shape, the analyses resulting in shells of the same height, namely cases a5 and b5 of Table 2 and Figure 5, predict a cap shape (namely, a shape with the same sign of the Gauss’ curvature within the entire shell area). In order to find out which parameter might be responsible for this discrepancy, we keep in mind the uncertainty about the thin metal film properties, which are known to strongly depend on its fabrication procedures and conditions. For example, the values of Al’s Young’s modulus found in the literature can vary between 8 GPa [32] and up to 74 GPa [33]. As it is suspected that one of the parameters defining the residual shell shape (bell or cap) might be the relative amount of reversible elastic strains released during unloading the stamping pressure, the sensitivity of the analysis results to the metal Young’s modulus was examined. This is presented in Figure 8, with the corresponding material and stamping parameters detailed in Table 3. A reduction in the Young modulus while keeping the yield stress unaltered results in an increase in the elastic part of deformation. Consequently, larger stamping peak deformations are required for achieving the center residual deflection of the real fabricated shell made out of plates with a smaller Young’s modulus. Similarly, it is expected that an increase in the yield stress will increase the stamping peak deformations required for achieving a desired residual center deflection.

**Figure 9:**
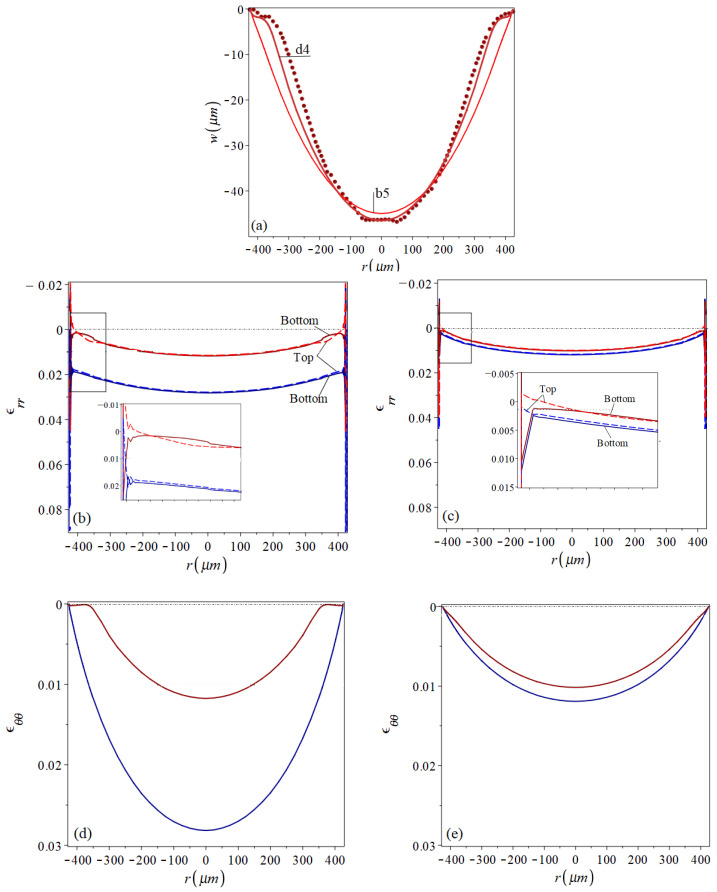
A comparison between (**a**) residual profiles with similar center deflection, as predicted for cases b5, d4 and the fabricated shell profile [9] (dark red dotted line); (**b**,**c**) radial and (**d**,**e**) tangential strain distributions at the stamping peak (shades of red lines) and at the final post-stamping stage (shades of blue lines) for cases d4 (**b**,**d**) and b5 (**c**,**e**). Dashed and solid lines represent strains on the sheet upper and lower surfaces, respectively. In addition, levels of assumed pre-strains are marked by black dotted lines and the level of zero strains is marked by black dash-dot lines.

Figure 8 reveals that the analyses based on lower values of Young’s modulus can be fit to better predict the residual actual shape. The best fit was achieved by case d4, with E=9 GPa and the assumed presence of pre-stamping stresses of 130 MPa. The comparison between the residual profiles of the shells d4 and b5, which are shown again in Figure 9a together with the profile of the real fabricated shell, provides an insight into how *E* affects the shapes stamped out of prestressed plates. The numerically predicted radial and tangential strain distributions under the stamping peak pressure and at the final post-stamping stage, for both analyses, are presented in Figure 9b–e. These distributions qualitatively agree with those presented in [38,40]. Figure 9b,c show that for both cases, the variation in the stamping radial strains along the radius is mild, excluding sharp changes over a very small region near the circumferential plate boundary. The large radial edge strains, which, unlike those over most of the plate region, have very different values on the top and bottom surfaces, indicate local bending. For both cases, the residual radial strains on the top and bottom surfaces reflect a residual convex curvature localized over a narrow inner ring near the perimeter. This is, however, more significant in the bell-shape shell d4.

While, in general, the radial strain pictures for both shells are qualitatively similar, the tangential strain distributions of the two shells, Figure 9d,e, reveal the difference between them. This is mainly expressed by the vanishing residual tangential strains over a ring of about 50μm width near the boundary of the d4 shell, which seem to be responsible for its bell shape. Over this region, the stamping tangential strains, which are much smaller than those developing over the inner plate region, are not large enough to bring the material of smaller *E* into the depth of plastic region. In the stiffer b5 case, the relatively uniform stamping strains are deep in the material’s elasto-plastic region. Consequently, the major part of the b5 plate experiences nonvanishing irreversible deformations that change its surface area all over, resulting in the residual cap shape.

*Role of the stamp–plate friction*. To illuminate the effect of the foam–plate friction during stamping, the fabrication of the d4 shell was analyzed using a friction coefficient of the values 0 and 1. The results of these analyses were compared to the results obtained with a friction coefficient of 0.1 (used for all other analyses), which were presented above. The three predictions were almost identical in terms of stamping as well as residual shapes, stresses and strains. The insensitivity of the process to the friction coefficient might be attributed to the high compressibility and small Poisson’s ratio of the foam material.

**Figure 10:**
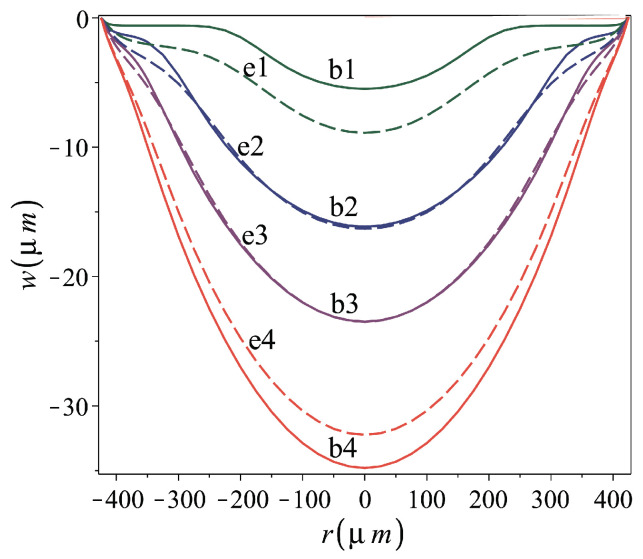
Effect of plate thickness on stamping and residual shape. Cases b1–b4 of constant thickness d=0.4μm and varying maximum stamping top foam displacement $v_{S}$ in solid lines; cases e1–e4 of varying thickness, *d*, and constant maximum stamping top foam displacement, $v_{S}=5$ mm, in dashed lines. Fabrication and material parameters for the analyzed cases are given in Table 2 and Table 4.

*Role of the flat stamped plate geometry*. The design of shells of desired dimensions involves suitable adjustments of the flat plate thickness and radius. Furthermore, as the flat circular plate is fabricated by the procedure described in Figure 2a,b, there is an uncertainty regarding its geometry. For example, the fact that the wall of the circular cavity opened by the back-side etching, Figure 2b, is not fully vertical [41] affects the actual value of the plate diameter. To examine the effect of the flat-plate geometry on the stamping process and results, the calculated residual profiles of micro shells of various thickness are shown in Figure 10. The thicknesses of shells e1–e4 and the characteristics of their stamping process are listed in Table 4. In the figure, the profile of 0.4μm thick shells b1–b4, details for which are given in Table 2, are also shown.

Table 4 implies that the residual-to-stamping-height ratio, $h_{0}/h_{0_{S}}$, decreases with the increase in the plate thickness ratio, d/R, namely that more irreversible plastic deformation is induced in the thinner stamped plates. The similarity between the residual profiles of e2 and b2 and those of e3 and b3 is apparent, and seems to be reflected by almost identical $h_{0}/h_{0_{S}}$ values, 0.636 and 0.647, for e2 and b2, respectively, and 0.781 for e3 and b3. However, to form the thicker shells e2 and e3, a larger punch top displacement, namely a larger stamping pressure, was required.

**Figure 11:**
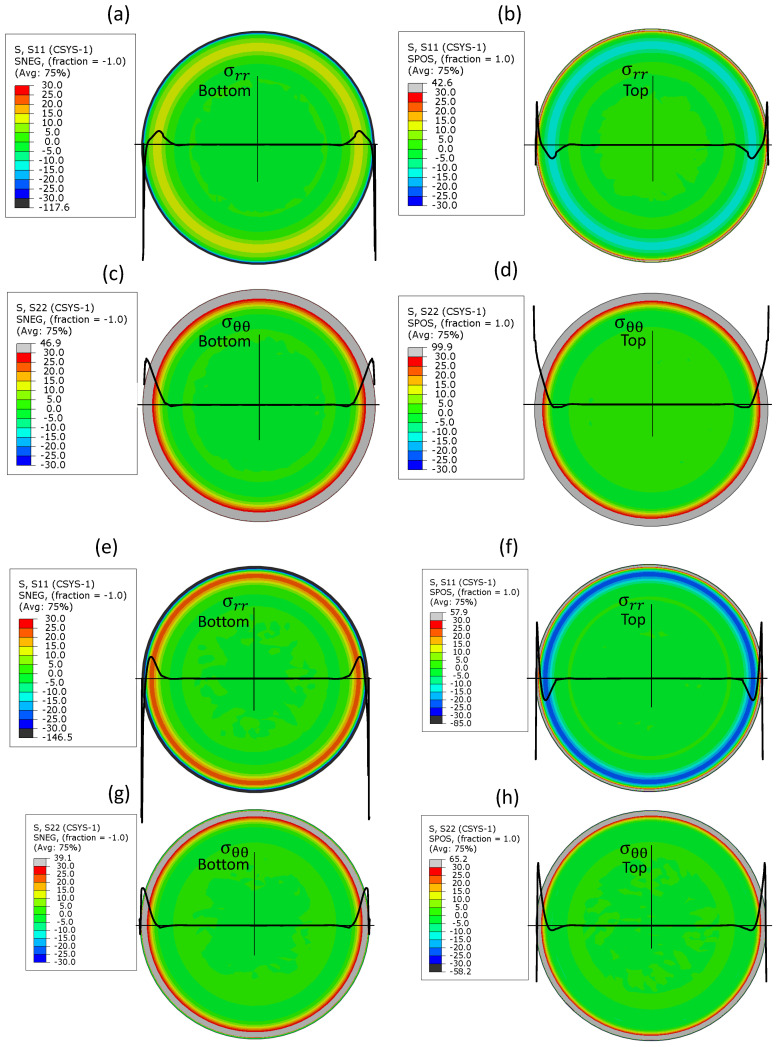
Post-stamping residual stresses (MPa) in (**a**–**d**) shell d4 and (**e**–**h**) shell b5. Radial residual stresses on the (**a**,**e**) lower and (**b**,**f**) upper surfaces. Tangential residual stresses on the (**c**,**g**) lower and (**d**,**h**) upper surfaces. Solid thick black lines on the colored maps represent the stress profile.

*Post-stamping residual stress distribution*. At the micro scale, the fabrication-related residual state of stresses may significantly affect the static and dynamic behavior of the structural element [5,10,20]; thus, its prediction is of paramount importance. The distributions of the radial and tangential post-stamping residual stresses in shells d4 and b5 are shown in Figure 11. Colored maps indicating the levels of the stresses on the upper and lower shell surfaces are accompanied by stress profiles along the diameter. The figure, illustrating probably one of the most important results of this work, indicates that cold forming redistributes the pre-stamping uniform state of stresses, yielding a stress relief over the internal region of the shell and residual stress concentration in the area in the vicinity of the plate clamped boundary. In this circumferential region, the opposite signs of the residual radial stresses on opposite shell surfaces reflect the presence of localized bending. The tangential residual stresses are uniformly distributed through the shell thickness. The width of the circumferential ring experiencing tangential residual stresses in the bell-shape shell d4 is larger than that in the cap-shape shell b5, which is made of a stiffer material.

## 6. Conclusions

An FE model was established for the analysis of a sheet cold forming process, which had been recently suggested for the microfabrication of bistable circular micro metal shells. Being based on moldless soft-punch stamping, the procedure results in shells of a shape that is noticeably dependent on the various geometric, material and process parameters. As the micro shell bistability is mainly conditioned by its curvature, the model was employed for a systematic study of the effect of the self-molding stamping procedure parameters on the resulting shell geometry. It was also employed for examining the state of the post-stamping residual stresses, which at the micro scale plays a role that cannot be ignored in the structural static and dynamic behavior. While qualitative and even reasonable quantitative agreement between the model prediction and previously presented experiment was obtained, our numerical parametric study had a primarily comparative character and was aimed at obtaining insight into how different stamping parameters affect the results.

It was found that the relations between the amounts of reversible elastic and irreversible plastic deformations, which accumulate during the stamping process, and their spatial distribution over the shell region, are of a paramount importance. They affect both the shell shape and state of residual stresses, and are, in turn, dominated mainly by the plate material parameters and the stamping pressure. Consequently, sheets of a smaller Young modulus and larger yield stress require a larger stamping peak deformation. Similarly, the presence of pre-stamping stresses may increase the springback effect. This influence becomes less pronounced in stamping processes inducing large irreversible inelastic deformations. Furthermore, it was found that the residual shell height, $h_{0}$, is mainly controlled by the deformation over the main internal region of the plate. The shell shape is controlled by the deformation of points near the plate perimeter. The bell shape is obtained as long as the residual tangential deformation of this near boundary region is very small. This is the case when the reversible elastic deformations form a large part of the total stamping deformation. This effect can be enhanced by smaller maximum stamping deflections, a smaller elastic modulus and/or a larger yield stress. As the large irreversible deformations initiating at the shell central region expand over its major part, which is typical for membrane behavior, a cap-like shell is obtained. In contrast, the stamping foam–plate friction was found to have a negligible influence.

Our results provide modeling tools to future designers and manufacturers of these kinds of systems. The present analysis approach can be extended to take into account more complicated contact conditions between the soft stamp and the metallic layer, as well as failure criteria for the plate material, in an attempt to better understand occasionally occurring rupture failure. Tracking the complete stamping (loading and unloading) path, in both experimental fabrication and numerical analysis, can provide an even wider-spectrum picture of the process.

While the present investigation was focused on modeling the fabrication of circular shells, the suggested self-molding soft-pad stamping fabrication procedure is suitable for the formation of 3D surfaces of various contour shapes. Properly adjusted analyses can be used for taking into account material anisotrpy, layered microstructre and varying thickness, such that material and geometric properties can be tailored to fabricate 3D micro surfaces of desired shapes and curvatures.

## Figures and Tables

**Table 1:** Storakers ([22,23]) foam material parameters, Equation (1), adopted through fitting between compression tests and Abaqus CAE analysis.

$\mu_{1}$, MPa	$\mu_{2}$, MPa	$\mu_{3}$, MPa	$\alpha_{1}$	$\alpha_{2}$	$\alpha_{3}$	ν
−0.083	0.160	−0.0000354	1.54	2.19	−1.43	0.01

**Table 2:** Parameters used in numerical analyses, providing the residual profiles presented in Figure 5. E=69 GPa and d=0.4μm.

Parameters/Case	a1	a2	a3	a4	a5	b1	b2	b3	b4	b5
$\sigma_{0}$ (MPa)	0	0	0	0	0	130	130	130	130	130
$v_{S}$ (mm)	2.0	2.5	3.0	4.0	5.0	2.0	2.5	3.0	4.0	5.0
$h_{0_{S}}$ (μm)	21.3	26.0	30.8	40.4	49.9	20.4	25.3	30.1	39.6	49.0
$h_{0}$ (μm)	9.9	18.4	24.9	35.9	46.1	5.5	16.1	23.5	34.8	45.0

**Table 3:** Parameters used in numerical analyses, providing the residual profiles presented in Figure 8. d=0.4μm.

Parameters/Case	c1	c2	c3	c4	c5	d1	d2	d3	d4
$\sigma_{0}$ (MPa)	0	0	0	0	0	130	130	130	130
*E* (MPa)	25	15	11	9	7.5	25	15	11	9
$v_{S}$ (mm)	4.3	4.3	4.3	4.3	4.3	5.3	5.3	5.3	5.3
$h_{0_{S}}$ (μm)	66.6	68.7	70.5	71.9	73.5	77.5	78.4	79.2	79.8
$h_{0}$ (μm)	57.2	53.1	49.0	45.5	41.5	67.7	61.5	54.4	46.5

**Table 4:** Parameters used for the plate thickness sensitivity study in the numerical analyses, providing the residual profiles presented in Figure 10. E=69 GPa, $\sigma_{0}=130$ MPa and $v_{S}=5$ mm.

Parameters/Case	e1	e2	e3	e4
*d* (μm)	1.2	1.0	0.8	0.6
$h_{0_{S}}$ (μm)	21.6	25.2	30.1	37.3
$h_{0}$ (μm)	8.9	16.3	23.5	32.2

## Data Availability

The original contributions presented in this study are included in the article. Further inquiries can be directed to the corresponding author.
